# Performance of the Biomark HD real-time qPCR System (Fluidigm) for the detection of nasopharyngeal bacterial pathogens and *Streptococcus pneumoniae* typing

**DOI:** 10.1038/s41598-019-42846-y

**Published:** 2019-04-24

**Authors:** Courtney P. Olwagen, Peter V. Adrian, Shabir A. Madhi

**Affiliations:** 1 Department of Science and Technology/National Research Foundation: Vaccine Preventable Diseases, Faculty Health Sciences, University of the Witwatersrand, Johannesburg, South Africa; 2 Medical Research Council: Respiratory and Meningeal Pathogens Research Unit, Faculty Health Sciences, University of the Witwatersrand, Johannesburg, South Africa

**Keywords:** Infectious-disease diagnostics, Biological sciences

## Abstract

Traditional qPCR assays for pneumococcal detection and serotype characterization require large sample volume, is expensive and labor intensive. We aimed to develop a quantitative nanofluidic Fluidigm assay to overcome some of these shortcomings. A quantitative Fluidigm assay was established to detect 11 bacterial pathogens, 55 pneumococcal serotypes and 6 serotypes of *H*. *influenzae*. The Fluidigm assay results were compared to conventional qPCR and culture. All reactions in the Fluidigm assay effectively amplified their respective targets with high sensitivity and specificity compared to qPCR. There was excellent concordance between qPCR and Fluidigm for detection of carriage prevalence (*kappa* > 0.75) and density (Rho > 0.95). Fluidigm identified an additional 7 (4.2%) serotypes over those detected by qPCR. There was a modest concordance between culture and Fluidigm for the majority of reactions detecting *S*. *pneumoniae* serotypes/serogroups (*kappa* > 0.6), with Fluidigm identifying an additional 113 (39.1%) serotypes. Discordant results between the three methods were associated with a low carriage density. The Fluidigm assay was able to detect common pneumococcal serotypes, *H*. *influenzae* serotypes, and other common nasopharyngeal bacterial organisms simultaneously. Deployment of this assay in epidemiological studies could provide better insight into the effect of PCV immunization on the nasopharyngeal microbiota in the community.

## Introduction

The human nasopharynx is a common ecological niche for several respiratory pathogens including *Streptococcus pneumoniae*, *Staphylococcus aureus*, *Moraxella catarrhalis*, and *Haemophilus influenzae*^1^. There is a paucity of data and conflicting findings on the interactions between these bacteria in the human nasopharynx^2–6^. Furthermore, most nasopharyngeal colonization studies have used non-quantitate culture based methods, with a lack of quantitative data available to assist in understanding the dynamics of interaction of these bacteria in the nasopharynx^7–9^. Recently, molecular quantitative PCR-based methods have been developed, which has assisted our understanding of bacterial nasopharyngeal colonization and its relationship to disease^9–12^. Serotyping of pneumococci by qPCR, however, has several shortcomings including large sample volumes needed to distribute across all reactions and it being labor intensive^10^.

Fluidigm is a nanofluidic automated real-time PCR system that relies on microfluidic technology in which dynamic arrays of integrated fluidic circuits (IFCs) are used. These IFC’s contain thousands of controlled valves and interconnected channels in which molecules of biological samples and reagents can be automatically mixed in a variety of patterns. The instrument uses an array of non-fluidic chips called dynamic arrays for qPCR, in which a typical chip format allows for 9 216 PCR reactions (96.96 chip format; 96 samples × 96 assays) in a single qPCR run (www.fluidigm.co.za)^13^. Other advantages of Fluidigm over standard qPCR include a larger number of reactions per plate, making it more cost-effective and less time-consuming. Further, IFCs not only reduce the reaction volume from 10 µL–20 µL down to 10 nL scale, but the technology allows for validations as well as increased throughput of qPCR reactions^13^.

We aimed to develop a novel nanofluidic real-time PCR (Fluidigm) assay that simultaneously detected and quantified 11 bacterial pathogens in 96 different samples (92 samples and 4 controls) in a single run. The assay included serotyping for 55 pneumococcal serotypes (16 individual serotypes and 39 serotypes in 13 groups) and six *H*. *influenzae* (serotypes a-f) serotypes. We compared the results of the Fluidigm assay to that of traditional qPCR for detection of bacterial colonization and pneumococcal serotyping in a cohort of PCV-vaccinated African children. Our previous work had compared qPCR to standard culture methods for 46 pneumococcal serotypes (14 individual serotypes and 32 serotypes among 10 serogroups) and 6 bacterial pathogens in a different cohort of children^10^. In these analyses, we expand on the latter by comparing the results from the Fluidigm assay to culture and qPCR.

## Results

From the initial 407 nasopharyngeal swab samples collected at the two study visits, 335 (82.3%) were available for subsequent molecular nanofluidic qPCR (Fluidigm) analysis (Supplementary Fig. 1). Of these available samples, 83.4% were Black-African and 49.1% were male. Data from the two study visits were combined for the main analysis as findings from both 9 and 16 months of age were similar.

### Performance of the Fluidigm

All reactions were effective in amplifying their respective targets with high sensitivity and specificity in the Biomark HD system (Fluidigm), with the efficiency of the reactions ranging from 89% to 105% (Supplementary Table 1). Within the linear dynamic range, the correlation coefficients (r^2^) of the reactions were 0.99, except for reactions detecting *H*. *influenzae* E, IS481 (*B*. *pertussis* and *B*. *holmesii*) and *S*. *pneumoniae* serotype 19A, were r^2^ was 0.98 (Supplementary Table 1). The limit of detection (LLD) for most of the reactions were equivalent >10 copies, with exception to primer/probes that detected *H*. *influenzae* serotype E, IS481 (*B*. *pertussis* and *B*. *holmesii*) and pneumococcal serotypes/serogroups 5, 6A/B/C/D, 6C/D, 13 and 21, in which the LLD was 10 fold less (>100 copies). Further, no cross-reactivity occurred, with all primer and probes being specific for their respective target. Lastly, for all respective reactions, the intra- and inter-assay variation was <0.1, while the accuracy was within ±0.1 (Supplementary Table 1).

### Detection of bacterial nasopharyngeal carriage by qPCR and Fluidigm

**Figure 1:**
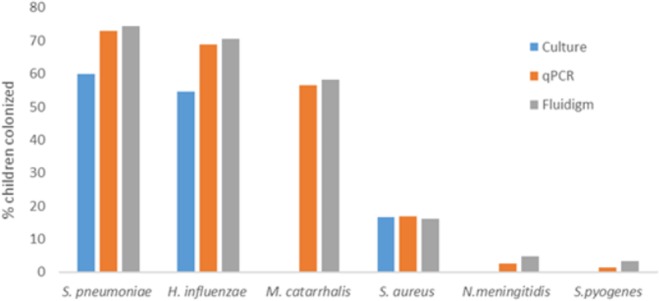
Prevalence of nasopharyngeal (NP) bacterial colonization in PCV-vaccinated, HIV-uninfected children as measured by culture, qPCR and Fluidigm. P-values of <0.05 was considered significant as determined by McNemar’s test.

**Figure 2:**
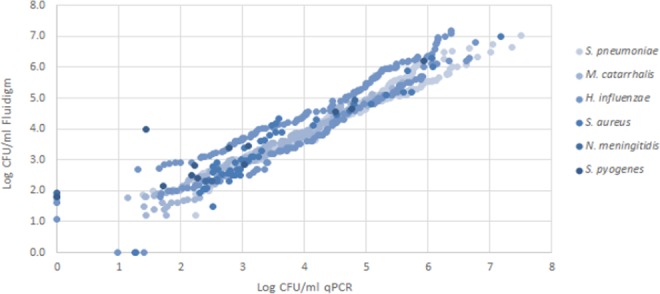
Density of colonization by common nasopharyngeal bacterial pathogens as determined by qPCR and Fluidigm.

There was excellent concordance between qPCR and Fluidigm for the detection of *S*. *pneumoniae* (*Kappa* = 0.98), *H*. *influenzae* (*Kappa* = 0.97), *M*. *catarrhalis* (*Kappa* = 0.98), *S*. *aureus* (*Kappa* = 0.98) and *S*. *pyogenes* (*Kappa* = 0.96); and a high concordance between qPCR and Fluidigm for the detection of *N*. *meningitides* (*Kappa* = 0.75; Fig. 1, Table 1). All additional bacteria detected by either qPCR or Fluidigm had estimated copy numbers of <10^2^ colony forming units (CFU)/ml per swab (Fig. 2) and thus discordance between the methods was strongly associated with the density of carriage. The sensitivity and specificity of the Fluidigm method compared to conventional qPCR were high with all reactions having a sensitivity >92% and a specificity of >99% (Table 1).

**Table 1:** Concordance between qPCR and Fluidigm for the detection of common nasopharyngeal pathogens in PCV-vaccinated, HIV-uninfected children.

Bacterial Target	qPCR	Fluidigm	P-value	Concordance (kappa)	Sensitivity (%)	Specificity (%)
*S*. *pneumoniae*	244(72.8)	246(73.4)	0.16	0.98	98.8	100
*H*. *influenzae*	230(68.7)	232(69.3)	0.32	0.97	98.7	99
*M*. *catarrhalis*	189(56.4)	192(57.3)	0.08	0.98	98.4	100
*S*. *aureus*	57(17)	55(16.4)	0.16	0.98	100	99.3
*S*. *pyogenes*	11(3.3)	12(3.6)	0.32	0.96	91.7	100
*N*. *meningitidis*	3(0.9)	5(1.5)	0.16	0.75	91.7	100

*B*. *bronchiseptica*, *B*. *parapertusis*, *B*. *pertussis*, *B*. *holmesii*, and *N*. *lactamica* were not tested by conventional qPCR and thus could not be compared to Fluidigm; however, the overall detection prevalence by Fluidigm was 0.3%, 0%, 0%, 0%, and 4.2% respectively. Further *H*. *influenzae* was not serotyped by conventional qPCR and thus could not be compared to Fluidigm; however, of the *H*. *influenzae* positive samples, 3.9% were serotype a, 1.3% were serotype b, 2.6% were serotype c, 1.3% were serotype d, 1.7% were serotype e, 4.3% were serotype f, and 83.6% were non-typable (*hinf*NT).

### Detection of bacterial nasopharyngeal carriage by culture and Fluidigm

There was excellent concordance between culture and Fluidigm assay for detection of *S*. *aureus* (*Kappa* = 0.86), and a modest concordance for detection of *H*. *influenzae* (*Kappa* = 0.63) and *S*. *pneumoniae* (*Kappa* = 0.59). The Fluidigm assay was more sensitive than culture in detecting *S*. *pneumoniae* (73.4% vs. 60%; p < 0.001) and *H*. *influenzae* (69.3% vs. 54.6%, p < 0.001; Fig. 1, Supplementary Table 2). Discordance for bacterial detection between culture and Fluidigm methods were associated with carriage density, with the majority of Fluidigm-positive but culture-negative samples being <10^4^ CFU/ml per swab, and the majority of culture-positive but Fluidigm negative samples reported as “scant” growth (<5 colonies/plate) on culture.

### Detection of pneumococcal serotype carriage by qPCR and Fluidigm

**Figure 3:**
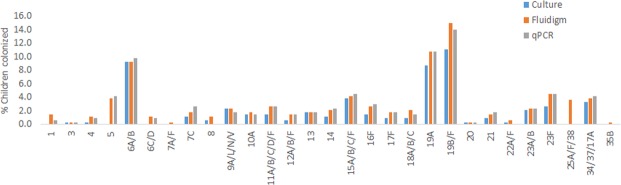
Prevalence of pneumococcal serotype colonization in PCV-vaccinated, HIV-uninfected children as measured by culture, qPCR and Fluidigm. P-values of <0.05 was considered significant as determined by McNemar’s test.

**Figure 4:**
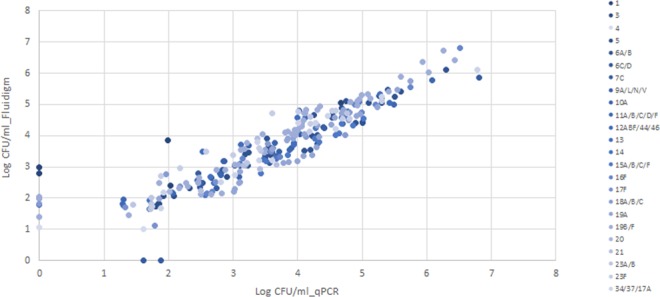
Density of colonization of pneumococcal serotypes as determined by qPCR and Fluidigm.

There was excellent concordance between qPCR and Fluidigm assays, with the majority of reactions detecting *S*. *pneumoniae* serotypes/serogroups (*Kappa* > 0.8) by both methods, with exception to serotype 1, for which the concordance was still high (*Kappa* = 0.75; Fig. 3, Table 2). Fluidigm was more sensitive in detecting serotypes/groups 1, 4, 10A, 18A/B/C, and 19B/F; while qPCR was more sensitive in detecting serotype 7C. In addition, the Fluidigm assay identified 7 (4.2%) additional serotypes compared to those identified by qPCR; and conversely, the qPCR method detected 2 (2.1%) additional serotypes not detected by Fluidigm. Discordant results between the methods were strongly associated with the density of carriage, with all additional pneumococcal serotypes/groups detected either by qPCR or Fluidigm having estimated copy numbers of <10^3^ CFU/ml per swab (Fig. 4). The sensitivity and specificity of the Fluidigm compared to qPCR assay were high; i.e. sensitivity >80% and specificity >99.3%; Table 2.

**Table 2:** Concordance between qPCR and Fluidigm for the detection of pneumococcal serotypes in PCV-vaccinated, HIV-uninfected children.

Serotype	qPCR	Fluidigm	P-value	Concordance (kappa)	Sensitivity (%)	Specificity (%)
*LytA*	244(72.8)	246(73.4)	0.16	0.98	98.8	100
1	3(0.9)	5(1.5)	0.16	0.75	95	100
3	1(0.3)	1(0.3)	—	1	100	100
4	3(0.9)	4(1.2)	0.32	0.86	80	100
5	13(3.9)	13(3.9)	—	1	100	100
6A/B	31 (9.3)	31 (9.3)	—	1	100	100
6C/D	2(0.6)	2(0.6)	—	1	100	100
7C	8(2.4)	6(1.8)	0.16	0.85	100	99.3
9A/L/N/V	8(2.4)	8(2.4)	—	1	100	100
10A	5(1.5)	6(1.8)	0.32	0.91	83.3	100
11A/B/C/D/F	9(2.7)	9(2.7)	—	1	100	100
12ABF/44/46	5(1.5)	5(1.5)	—	1	100	100
13	6(1.8)	6(1.8)	—	1	100	100
14	7(2.1)	7(2.1)	—	1	100	100
15A/B/C/F	14(4.2)	14(4.2)	—	1	100	100
16F	9(2.7)	9(2.7)	—	1	100	100
17F	6(1.8)	6(1.8)	—	1	100	100
18A/B/C	6(1.8)	7(2.1)	0.32	0.93	85.7	100
19A	36(10.7)	36(10.7)	—	1	100	100
19B/F	48(14.3)	50(14.9)	0.08	0.96	94	100
20	1(0.3)	1(0.3)	—	1	100	100
21	5(1.5)	5(1.5)	—	1	100	100
23A/B	8(2.4)	8(2.4)	—	1	100	100
23F	15(4.5)	15(4.5)	—	1	100	100
34/37/17F	13(3.9)	13(3.9)	—	1	100	100

Serotypes/serogroups 7A/F, 8, 22A/F, 25AF/38 and 35B were only tested by Fluidigm, with an overall detection prevalence of 0.3%, 0.9%, 0.6%, 2.7% and 0.3%, respectively.

### Detection of pneumococcal serotype carriage by culture and Fluidigm

Generally, there was high concordance between culture and the Fluidigm assay for detecting *S*. *pneumoniae* serotypes/serogroups. Culture identified 21 (10.9%) serotypes/serogroups not detected by the Fluidigm assay, and conversely the Fluidigm assay identifying as additional 113 (39.1%) serotypes not identified by culture methods (Fig. 3, Supplementary Table 3). This included higher detection prevalence by the Fluidigm assay for serotypes/serogroups 1 (1.5% vs. 1%; *p* = 0.03), 5 (8.9% vs. 0%; *p* = 0.003), 16 (2.7% vs. 1.5%, *p* = 0.05), 18A/B/C (1.8% vs. 0.9%, *p* = 0.05), 19B/F (14.9% vs. 11%, *p* = 0.002), 23F (4.5% vs. 2.7%, *p* = 0.03), and 25A/25F/38 (3.6% vs. 0%, *p* = 0.005). The additional serotypes detected only by Fluidigm mostly had densities <10^4^ CFU/ml per swab. Furthermore, the majority of the additional serotypes detected by Fluidigm were carried concurrently with other pneumococcal serotypes, of 89% were a non-primary colonizing serotype (Supplementary Table 4). The sensitivity of the Fluidigm assay compared to culture were high for the majority of reactions; mainly having sensitivity >80%, with the exception of serotype 10A (60%) and serogroup 23A/B (71%). All Fluidigm reactions had high specificity (>95%) compared to culture (Supplementary Table 3).

### Density of bacterial nasopharyngeal carriage as determined by qPCR and Fluidigm

There was an excellent correlation (Rho > 0.95) between qPCR and Fluidigm for measuring the density of colonization (Figs 2 and 4), with no significant differences observed between the two methods for all bacterial pathogens and pneumococcal serotypes (Table 3).

**Table 3:** Density of bacterial nasopharyngeal carriage as determined by qPCR and Fluidigm.

Bacteria	Density detected by qPCR	Density detected by Fluidigm	P-value
*S*. *pneumoniae*	4.54(4.37–4.70)	4.39(4.24–4.54)	0.49
*H*. *influenzae*	4.38(4.21–4.55)	4.56(4.39–4.73)	0.18
*M*. *catarrhalis*	3.38(3.23–3.52)	3.29(3.15–3.44)	0.37
*S*. *aureus*	3.48(3.17–3.79)	3.41(3.08–3.75)	0.13
*S*. *pyogenes*	3.09(2.14–4.03)	3.68(3.04–4.32)	0.11
*N*. *meningitides*	3.24(−0.13–6.61)	2.96(0.20–5.73)	0.18
**Pneumococcal serotypes/serogroups**			
1	2.90(−0.92–6.71)	3.76(0.46–7.07)	0.22
3	3.55	3.66	—
4	2.85(−0.27–5.97)	2.30(−1.20–7.20)	0.79
5	2.52(2.01–3.02)	2.46(2.0–2.91)	0.09
6A/B	4.25(3.88–4.61)	4.07(3.77–4.36)	0.056
6C/D	4.05(−1131–19.42)	3.60(−5.11–12.30)	0.54
7C	3.49(1.73–5.25)	3.27(1.89–4.67)	0.33
9A/L/V/N	3.31(2.52–4.10)	3.23(2.52–3.95)	0.16
10A	3.23(2.47–3.98)	3.62(2.64–4.1)	0.17
11A/B/C/D/F	3.29(2.03–4.56)	3.21(2.16–4.27)	0.51
12A/12B/12F/44/46	3.38(1.39–5.38)	3.58(1.76–5.40)	0.19
13	3.96(2.85–5.06)	4.03(3.13–4.94)	0.82
14	3.40(2.06–4.74)	2.93(2.37–3.48)	0.29
15A/B/C/F	3.81(2.93–4.68)	3.54(3.01–4.07)	0.075
16F	4.50(3.43–5.56)	4.23(3.01–5.44)	0.14
17F	2.54(1.55–3.53)	2.55(1.67–3.44)	0.88
18A/B/C	3.84(2.39–5.28)	3.84(2.96–4.73)	0.98
19A	3.82(3.48–4.17)	3.75(3.4–4.10)	0.26
19B/F	3.87(3.49–4.24)	4.14(3.78–4.51)	0.66
20	ND	ND	
21	3.38(2.23–4.53)	3.13(2.58–3.68)	0.55
23A/B	2.92(−0.76–4.92)	2.58(0.31–4.86)	0.16
23F	3.64(3.12–4.16)	3.98(3.44–4.51)	0.181
34/37/17A	3.90(3.08–4.72)	3.41(2.88–3.94)	0.059

Numbers are geometric mean densities (95% Confidence intervals).

P-values of 0.05 were considered significant as determined by a paired student t-test.

ND, not done; too few variables to calculate.

## Discussion

A reliable molecular nanofluidic real-time PCR (Fluidigm) system was established to simultaneously detect 11 bacterial pathogens, 55 pneumococcal serotypes (16 individual serotypes and 39 serotypes in 13 groups) and 6 serotypes of *H*. *influenzae* (serotypes a-f) in a single run^14^. There was high concordance between qPCR and Fluidigm, including for identifying bacterial colonization, density of bacterial targets and pneumococcal serotypes, with discordant results mainly associated with samples in which the carriage density was low (<10^2^ CFU/ml per swab).

Discordance between the methods included the qPCR method detecting an additional 3 bacterial species and 2 pneumococcal serotypes above those detected by Fluidigm, and conversely the Fluidigm assay detecting an additional 11 bacterial species and 7 pneumococcal serotypes above those detected by qPCR. Although most of these differences could not be confirmed by culture, due to the additional pneumococcal serotypes detected being non-primary (second or third colonizing serotypes; Supplementary Fig. 3) colonizers, one additional serogroup 19B/F isolate detected by Fluidigm and one additional serotype 7C isolate detected by qPCR were concordant with culture results. These small differences (<5%) in the detection of targets between qPCR and Fluidigm methods are, however, expected based on Poisson’s distribution that the assays had a 95% probability of detecting their respective targets with reasonable certainty^14^. This was further supported by the discordant readouts between the methods mainly being associated with samples where the density of carriage was very low (<10^2^ CFU/ml). Furthermore, the higher yield on the Fluidigm assay could be due to a pre-amplification step that selected for targets in this assay (and not the qPCR assay).

While culture remains the referent standard for bacterial carriage and pneumococcal serotype characterization studies, these assays lack quantitative data which impose limitations on our understanding of the association between nasopharyngeal carriage and its relation to disease^15–19^. Recently, several molecular qPCR methods have been developed to overcome the above shortcoming of conventional culture assays. Nevertheless, most molecular methods are still limited by the large sample volume required to distribute across all reactions, cost (approximately $ 1.85 per sample per reaction in duplex) and processing multiple qPCR reactions being labor intensive and time-consuming^10^. Further, there is a limited number of quantitative studies that have concurrently described colonization of a diverse number of bacterial pathogens and pneumococcal serotypes.

Our Fluidigm assay addressed some of the challenges posed by qPCR assays, including analyzing a larger number of reactions per plate making the assay more cost-effective (approximately $ 0.37 per sample per reaction), less time-consuming and less labor intensive (9216 reactions per one Fluidigm plate versus 96 reactions per one qPCR plate). Further, IFCs not only reduces the reaction volume from 10 µL–20 µL down to 10 nL scale, but the technology allows for validations as well as increased parallelism and throughput of qPCR reactions^13^.

Notably, the Fluidigm assay was more sensitive in detecting serotype 5 (3.9% vs. 0%) and serogroup 25AF/38 (3.6% vs. 0%) compared to culture (Fig. 3). Half of the isolates typed as serogroup 22AF/38 were not identified on culture, while the other half were commonly found as non-primary colonizers (Supplementary Fig. 2), which might explain them not being identified by culture methods. The majority of serotype 5 isolates (12/13; 92%) were commonly isolated as non-primary serotypes, raising the possibility of false positive readouts from the Fluidigm assay. These results were consistent with findings when we analyzed using the qPCR compared to culture assay^10^. This is of particular importance for serotype 5 since the primers used also detect *S*. *mitis in Silio*. Nevertheless, we recommend that results from serotype 5 and serogroup 22AF/38 are interpreted with caution and further investigation is warranted.

The Biomark HD system in a new instrument that has only been used to serotype pneumococcus by one other group; however, their assay differed in that they used Evagreen (a dsDNA-binding dye) chemistry instead of TaqMan probes to quantify bacterial carriage. Further, the study by Dhoubhadel *et al*. was designed to only detect 50 pneumococcal serotypes (17 individual serotypes and 33 serotypes in 12 groups) and did not include any additional bacterial pathogens^20,21^. The performance of our assay was, however, comparable to that described by Dhoubhadel *et al*.

Limitations of our study include that Fluidigm assay was unable to discriminate between all pneumococcal serotypes within some serogroups. Also, although the Fluidigm assay included additional pneumococcal serotypes and other bacterial targets compared to those investigated for by qPCR^10,11^, we did not test for all known pneumococcal serotypes. This should be considered for future studies. Further, the *LytA* gene was chosen to detect overall pneumococcal colonization; however, recent studies reported other isolates within the *S*. *mitis* group also harbor *LytA*^22^. No serotype was discernible for a small percentage (7.9%) of LytA positive samples detected by Fluidigm in our study, suggesting they were either non-typable or belonged to one of the serotypes not included in our assay. Alternately, these could have been *LytA* positive non-pneumococcal species that were being identified. Future studies on pneumococcal colonization using qPCR or Fluidigm should consider including an alternative gene such as *Xisco*, which is purportedly only present in *Streptococcus pneumoniae*^23^. As an initial step for gene expression on the Biomark HD Fluidigm system manufacturers recommends Specific target amplification (STA) for each sample to be done by combining all primers in a pool. Due to the high similarity between some pneumococcal primers, non-specific binding of primers to each other resulted (results not shown) in cross-specificity between some of the pneumococcal serotyping targets. This was addressed by separating primers into two separate pools (Supplementary Table 5), and then combing the STA products from the two assays. Careful monitoring and optimization of the Fluidigm assay are thus needed in future studies, especially if the assay is to be expanded with additional serotypes.

In conclusion, we established a Fluidigm assay that was highly sensitive and specific. Using the Fluidigm assay enabled simultaneous detection of nasopharyngeal colonization by common pneumococcal serotypes, *H*. *influenzae* serotypes, and other common nasopharyngeal bacterial pathogens; and provided quantitative data in a single run.

## Material and Methods

### Study population

Archived nasopharyngeal swab samples collected from a PCV7-vaccinated cohort of HIV-uninfected infants in Soweto, South Africa were retrospectively analyzed. The study cohort has been previously described^24,25^. Briefly, infants enrolled between April 2005 and June 2006 were 6–12 weeks old at enrolment and included participants who were both HIV-exposed-uninfected (HEU) and HIV-unexposed. These infants were scheduled to receive three doses of PCV7 (i.e. Prevnar®; Wyeth Vaccines, NJ, USA) at 6, 10 and 14 weeks of age^25,26^. PCV immunization was introduced into the public EPI program in May 2009 and thus, during the course of the study, PCV immunization of children in Soweto (birth cohort 28,000 per annum) was limited mainly to study-participants (approximately 600)^27^. As described, standard culture methods were used to culture samples for *S*. *pneumoniae* and the Quellung method was undertaken for serotyping^28^.

Nasopharyngeal (NP) swabs were collected at several time intervals, including at 9 and 16 months of age. Swabs were stored in skim milk-tryptone-glucose-glycerol (STGG) transport media at the Respiratory and Meningeal Pathogen Research Unit (RMPRU) in South Africa^29^. Samples were screened previously for *S*. *pneumoniae*, *H*. *influenzae*, *S*. *aureus*, *M*. *catarrhalis*, *S pyogenes*, and *N meningitides* by qPCR^11^, and pneumococcal serotyping was done by qPCR on all samples that tested positive for *S*. *pneumoniae* as described^30^. In this study, we now developed and evaluated a Fluidigm assay which screened for an additional 9 serotypes/serogroups (7A/F, 8, 22A/F, 25AF/38 and 35B) not included in our earlier qPCR assay, as well as investigated for presence of additional bacteria (*Bordetella bronchiseptica*, *Bordetella parapertusis*, *Bordetella pertussis*, *Bordetella holmesii,* and *Neisseria lactamica*. The Fluidigm assay was also designed to serotype *H*. *influenzae*. The additional serotypes included in the Fluidigm assay were chosen based on the most frequently isolated non-vaccine serotypes associated with colonization in South Africa as detected by culture methods, at the time of study design.

### Bacterial and pneumococcal reference isolates

Control strains for the pneumococcal serotypes (1, 2, 3, 4, 5, 6A, 6B, 6C, 6D, 7A, 7F, 7C, 8, 9A, 9L, 9N, 9V, 10A, 11A, 11B, 11C, 11D, 11F, 12A, 12B, 12F, 13, 14, 15A, 15B, 15C, 15F, 16A, 16F, 17A. 17F, 18A, 18B, 18C, 19A, 19B, 19F, 20, 21, 22A, 22F, 23A, 23B, 23F, 25A, 25F, 34, 35B and 37), *H*. *influenzae* serotypes (serotypes a-f), *S*. *aureus*, *M*. *catarrhalis*, *N*. *meningitidis* and *S*. *pyogenes* were obtained from the National Institute for Communicable Diseases (NICD). Additional isolates for *B*. *bronchiseptica* (ATCC® 4617), *B*. *parapertusis* (ATCC® 15311), *B*. *pertussis* (ATCC® 2397) and *N*. *lactamica* (ATCC® 23970) were purchased from Davies Diagnostics (South Africa), while an isolate for *B*. *holmesii* (ATCC® 51541) was purchased from LGC standards South Africa. DNA from these strains were used to optimize the Fluidigm reactions and as positive controls.

### DNA extraction

Total nucleic acid was automatically extracted from nasopharyngeal swab samples stored in STGG, using the NucliSens® easyMAG® extraction system (BioMérieux, Marcy l′Etoile, France), according to manufactures instructions. Similarly, total nucleic acid was also extracted from pneumococcal reference strains (positive control strains) grown in Todd-Hewitt broth supplemented with 5% yeast. *S*. *aureus*, *M*. *catarrhalis*, *S*. *pyogenes*, *N*. *meningitidis,* and *N*. *lactamica* reference strains were grown in Todd-Hewitt broth alone, *H*. *influenzae* serotypes a-f were grown in Brain-Heart infusion (BHI) broth and Bordetella species were grown in potato broth. Extracted DNA from samples and reference strains were stored at −20 °C.

### Real-time qPCR multiplex assay

Target DNA was previously pre-screened for *S*. *pneumoniae*, *M*. *catarrhalis*, *H*. *influenzae*, *S*. *aureus*, *N*. *meningitidis* and *S*. *pyogenes* as described^10,11^. All *LytA* positive samples (Cq < 35) were further molecularly serotyped for PCV7 serotypes/groups (4, 6A/B, 9A/L/N/V, 14, 18A/B/C, 19B/F and 23F) and non-vaccine serotypes/groups (1, 3, 4, 5, 6C/D, 10A, 11A/B/C/D/F, 12A/B/F, 13, 15A/B/C/F, 16F, 17F, 19A, 20, 21, 23A/B and 34/37/17A) as described^10^.

### Fluidigm assay

ABI primer express software package, version 3.0 (Applied Biosystems, ABI, Foster City, USA) was used to design oligonucleotide primers and FAM dye-labelled MGB probes for additional targets not included in the multiplex qPCR assay. Primer and probe sequences used in the Fluidigm assay are described in Table 4. GAPDH and BexA were included to confirm the efficiency of the DNA extraction and to confirm the presence of non-typable *H*. *influenzae*, respectively. The Fluidigm method was unable to discriminate between some pneumococcal serotypes within a particular serogroup due to the genotypic similarities between the capsule loci of certain serotypes (including serotypes: 6A/B, 6C/D, 7A/F, 9A/L/N/V, 11A/B/C/D/F, 12A/B/F/44/46, 15A/B/C/F, 18A/B/C, 19B/F, 22A/F, 23A/B, 25A/25F/38 and 34/37/17A); however, the method was able to identify serotypes 1, 3, 4, 5, 7C, 8, 10A, 13, 14, 16F, 17F, 19A, 20, 21, 23F and 35B individually.

**Table 4:** Oligonucleotide primers and probe^a^ sequences for quantitative molecular Fluidigm detection of respiratory pathogen.

TargetName	Primer/Probename	Forward primer	Reverse primer	Probe	Reference
Streptococcus pneumoniae	lytA	TCTTACGCAATCTAGCAGATGAAGC	GTTGTTTGGTTGGTTATTCGTGC	TTTGCCGAAAACGCTTGATACAGGG	McAvin*et al*.^31^
Haemophilus influenzae	IGA	CAAAATTGCCAAGATTAAATGCTT	TGCTCGCCATACTGCACA A	CCTGCGGTTAAACC	This study
Haemophilus influenzaetype A	HiA	GCAACCATCTTACAACTTAGCGAATAC	GGTCTGCGGTGTCCTGTGTT	AAGTGAAGCATGTCGCCATTCGTCCA	Maaroufi *et al*.^32^
Haemophilus influenzaetype B	HiB	TGTTCGCCATAACTTCATCTTAGC	CTTACGCTTCTATCTCGGTGATTAATAA	CACAAAACTTCTCATTCTTCGAGCCTA	Maaroufi *et al*.^32^
Haemophilus influenzaetype C	HiC	TCTGTGTAGATGATGGTTCAGTAG	TTAGGATATTTACGCTGCCATT	TGCAGCTAAGATTATT	Maaroufi *et al*.^32^
Haemophilus influenzaetype D	HiD	TATTGATGACCGATACAACCTGTTTAAA	CCAGAAATTATTTCTCCGTTATGTTGA	AATGGTTGTAAAACTCTTCT	Maaroufi *et al*.^32^
Haemophilus influenzaetype E	HiE	GTTGAAAACAAACCGCACTTT	ATCTTTAATTACCAGATCCCTTTCAT	AACGAATGTAGTGGTAGTTAGA	Maaroufi *et al*.^32^
Haemophilus influenzaetype F	HiF	GGATAATCAAATACCACATTGGCTTA	GTAGATTAGCCTCAATAACATGTGAATTAA	TCATCGTGAGATCATTGATCACGAT	Maaroufi *et al*.^32^
BexA	BexA	CTGAATTRGGYGATTATCTTTATGA	ACAATCAAAYTCAACHGAAAGHGA	AGGGATGAAAGCYCGRCTTGCAT	Maaroufi *et al*.^32^
Staphylococcus aureus	SA	GCTCAGCAAATGCATCACAAA	CACTATATACTGTTGGATCTTCAGAACCA	AGATAACGGCGTAAATA	This Study
Moraxella catarrhalis	MCAT	CCGCTTTTACAACCACTGCTT	TGTATCGCCTGCCAAGACAA	CAGCTGTTAGCCAGCC	This Study
Streptococcus pyogenes	SPY	GCACTCGCTACTATTTCTTACCTCAA	GTCACAATGTCTTGGAAACCAGTAAT	CCGCAACTCATCAAGGATTTCTGTTACCA	CDC 2008
Neisseria meningitidis	SodC	GCACACTTAGGTGATTTACCTGCAT	CCACCCGTGTGGATCATAATAGA	CATGATGGCACAGCAACAAATCCTGTTT	Thomas *et al*.^33^
Neisseria lactamica	LacZ	TTGCCCGAGAACCATTGTATC	GCGGTTCTTATCACGTTCTATATTTG	TATTGGAGCGGACTAAA	ThisStudy
Bordetella pertusis and Bordetella holmesii	IS481	CAAGGCCGAACGCTTCAT	GAGTTCTGGTAGGTGTGAGCGTAA	CAGTCGGCCTTGCGTGAGTGGG	Tatti*et al*.^34^
Bordetella holmesii	hIS1001	GGCGACAGCGAGACAGAATC	GCCGCCTTGGCTCACTT	CGTGCAGATAGGCTTTTAGCTTGAGCGC	Tatti*et al*.^34^
Bordetella parapertusis	pIS1001	TCGAACGCGTGGAATGG	GGCCGTTGGCTTCAAATAGA	AGACCCAGGGCGCACGCTGTC	Tatti*et al*.^34^
Bordetella pertusis, Bordetella parapertusis and Bordetella bronchiseptica	PtxS	CGCCAGCTCGTACTTC	GATACGGCCGGCATT	AATACGTCGACACTTATGGCGA	Tatti*et al*.^34^
Streptococcus pneumoniae serotype 1	1	CGTGCGGTAATTGAAGCTATGA	TGTGGCCCCAGCAACTCT	TGCTTGCCCTTGTATAGGGT	Azzari*et al*.^35^
Streptococcus pneumoniae serotype 3	3	GGTCAGCAGAAAGTATGCATTGG	TCGTTTATCCAGGGTCTGATGA	TATTGGATGTGGTTTATCGTGAAGA	Azzari*et al*.^35^
Streptococcus pneumoniae serotype 4	4	TGGGATGACATTTCTACGCACTA	CCGTCGCTGATGCTTTATCA	TCCTATTGGATGGTTAGTTGGTGA	Azzari*et al*.^35^
Streptococcus pneumoniae serotype 5	5	TTACGGGAGTATCTTATGTCTTTAATGG	CAGCATTCCAGTAGCCTAAAACTAGA	TTGTCTCAGCAACTCTATTTGGCTGTGGG	Azzari*et al*.^35^
Streptococcus pneumoniae serogroup 6A/B/C/D	6A/B/C/D	AAGTTTGCACTAGAGTATGGGAAGGT	ACATTATGTCCRTGTCTTCGATACAAG	TGTTCTGCCCTGAGCAACTGG	Azzari*et al*.^35^
Streptococcus pneumoniae serogroup 6C/D	6C/D	TTGGGATGATTGGTCGTATTAG	CTCTTCAATTAGTTCTTCAGTTCG	CCACGCAATTCGCCATC	Azzari*et al*.^35^
Streptococcus pneumoniae serogroup 7A/F	7A/F	GATGGCATGTGGCAAACCA	TTTGCCCTCCTTAATCATTTCAC	TTGGCTATCGGCATGGTGGT	Azzari*et al*.^35^
Streptococcus pneumoniae serotype 7C	7C	CGTCAGGAATAGGTGCAATCTCT	TGAAATTCCAAGCGAAGCAA	TTC ATCTATTGGTTCTTATGGTGTT	ThisStudy
Streptococcus pneumoniae serotype 8	8	CCACTCATCAGTTTCCCATATGTTT	TCAATAATTGAAGAAGCGAACGTT	TGATGGCAGATGGGTTGGGACGAG	Azzari*et al*.^35^
Streptococcus pneumoniae serogroup 9A/L/N/V	9A/L/N/V	TGGAATGGGCAAAGGGTAGTA	TCGGTTCCCCAAGATTTTCTC	TTAATCATGCTAACGGCTCATCGA	Azzari*et al*.^35^
Streptococcus pneumoniae serotype 10A	10A	CCTCTCCTATCAACTATTACTCATTATACTACCT	AATAACCATAAGTCCCTAGATCATTCAAAG	TCATTACAACTCCCTATGTGACACGGGTCTTTT	Azzari*et al*.^35^
Streptococcus pneumoniae serogroup 11A/B/C/D/F	11A/B/C/D/F	ACCGCATTTCTTATCGCACTATATT	TCTCCTTACCATCAAACATGTTAATCA	TGAATCAGTCTGACCGTTT	ThisStudy
Streptococcus pneumoniae serogroup 12ABF/44/46	12A/12B/12F/44/46	GATTATTCGCTTGCCTCTTCATG	ATAGCCGAAATAAGCTTTCCAGAA	ATTTGTAAGCGGACGTGCGATT	Azzari*et al*.^35^
Streptococcus pneumoniae serotype 13	13	TCGGATTTAGTAGTAACCCCATTGA	TTCTTGATTGAGGATGCATTTCC	AGTAGTAAGAGATCATATTCAAG	ThisStudy
Streptococcus pneumoniae serotype 14	14	CGACTGAAATGTCACTAGGAGAAGAT	AATACAGTCCATCAATTACTGCAATACTC	TGTCATTCGTTTGCCAATACTTGATGGTCTC	Azzari*et al*.^35^
Streptococcus pneumoniae serogroup 15A/B/C/F	15A/B/C/F	TTGAATCAGGTAGATTGATTTCTGCTA	CTCTAGGAATCAAATACTGAGTCCTAATGA	CTCCGGCTTTTGTCTTCTCTGT	Azzari*et al*.^35^
Streptococcus pneumoniae serogroup 16F	16F	GCAACTGGTATTTTTGATATTGGAGAA	CAAAGGAATGCCATGCCATA	AAAATGCTAACTTCGTTGGAGG	ThisStudy
Streptococcus pneumoniae serotype 17F	17F	GTAAAGATTTCATGTCCTATAAGGGAGAA	AGGCGTCCCTGTTTATGAGAAG	TTGTACATGGTCTGGATTT	ThisStudy
Streptococcus pneumoniae serogroup 18A/B/C	18A/B/C	CCTGTTGTTATTCACGCCTTACG	TTGCACTTCTCGAATAGCCTTACTC	AACCGTTGGCCCTTGTGGTGGA	Azzari*et al*.^35^
Streptococcus pneumoniae serotype 19A	19A	TTCGACGACGTATCAGCTTCA	TCATTGAGAGCCTTAACCTCTTCA	ACCCAAAACGGTTGACGCATTATACT	Azzari*et al*.^35^
Streptococcus pneumoniae serogroup 19B/F	19B/F	GGTCATGCGAGATACGACAGAA	TCCTCATCAGTCCCAACCAATT	ACCTGAAGGAGTAGCTGCTGGAACGTTG	Azzari*et al*.^35^
Streptococcus pneumoniae serotype 20	20	AAAGATACTGGCTGAGGAGCTATCTATT	AGTCAAAAGTACTCAACCATTCTGATATATTC	AGGATAAGGTCTACTTTGTGGGAGTTC	Azzari*et al*.^35^
Streptococcus pneumoniae serotype 21	21	CCATTTGAAGGACCAGTTGTTG	AAAAAGCCACTATCAGGAATACCAA	AATGGCATTGCTTCGTAAA	ThisStudy
Streptococcus pneumoniae serotype 22A/F	22A/F	TCTATTAAATAACCCATTGGAATTGAAACG	TCGCAATTGAAGACCACATAAACTG	TCCGTAAT”T”CGCTTATGGGCACATTCTCCA	Azzari*et al*.^35^
Streptococcus pneumoniae serogroup 23A/B/F	23A/B/F	GGTGGACTTTCCGATGCAA	CACTGTCAACAAAAATGAGGTAATCTC	AAATGTCGGTATAGATAAAG	ThisStudy
Streptococcus pneumoniae serotype 23F	23F	TGCTATTTGCGATCCTGTTCAT	AGAGCCTCCGTTGTTTCGTAAA	TTTCTCCGGCATCAAACGTTAAG	Azzari*et al*.^35^
Streptococcus pneumoniae serogroup 25A/25F/38	25A/25F/38	GTCTTACGTAGAACCTCTCTGGATGA	TGGTCCTACAAGCGACATGTG	TTGCCACAGATTTGGAATATTTTGGTCGG	ThisStudy
Streptococcus pneumoniae serogroup 34/37/17A	34/37/17A	GGATACTATGTACGAACAGATGGACTTG	CTCACTAACTCGCCCGAATAAAC	CCGACTATACTCCATTTGA	ThisStudy
Streptococcus pneumoniae serotype 35B	35B	GCATGGAGGTGGAGCATACA	TGTAAAGACTGCACAACTCGATATAAAA	CAATTTAAACAATATTAGTAAAGCGCAGGTCAAGCAAA	Azzariet al.^35^

Probe^a^: Minor Grove Binding (MGB) FAM labelled TaqMan probes.

### Pre-amplification of DNA for Fluidigm

Specific target amplification (STA) was done per manufactures recommendations as the initial step (pre-amplification of DNA) for the Biomark HD system (Fluidigm). Briefly, two separate pools containing 24X TaqMan assays in each pool as described in Supplementary Table 5, were prepared by mixing forward and reverse primers and diluted with 10 mM Tris/HCL and 0.1 mM EDTA (pH 8) to give a final concentration of 200 nM of each primer. PCR reactions were carried out for each STA pool in 5 µl volumes each containing 2.5 µL TaqMan PreAmp Master Mix (Fluidigm, CA, USA), 1.25 µL of pooled assay and 1.25 µL of DNA. Reactions were amplified with the T100 Thermal Cycler (Bio-Rad Laboratories, CA, USA) and cycling conditions included an initial activation at 95 °C for 10 minutes followed by 14 two-step cycles (denaturation at 95 °C for 15 seconds and annealing/extension at 60 °C for 4 minutes). STA products from the two PCR reactions were combined and diluted 1:5 with 10 mM Tris/HCL and 0.1 mM EDTA (pH 8).

### Real-time qPCR using the Biomark HD System (Fluidigm)

Fluidigm was carried out with 96.96 dynamic arrays (Fluidigm Corporation, CA, USA) according to manufactures instructions. 10X assay mixture and sample pre-mix were prepared, with each assay being prepared in duplicate (making up a total of 96 assays). Briefly, each 10X assays contained 2.5 ul 20X TaqMan gene expression assay (Fluidigm), prepared by mixing forward primer, reverse primers and probe, and 2.5 ul 2X Assay Loading Reagent (Fluidigm). The final concentration (at 10X) for each primer was 9 μM and 2 μM for the probe. Sample pre-mix was made by combining 2.5 ul TaqMan Universal PCR Master Mix (2X (Life Technologies, 0.25 ul 20X GE Sample Loading Reagent (Fluidigm PN 100–7610) and 2.5 ul pre-amplified cDNA (diluted STA product containing a negligible concentration of carried-over STA primers) for each of the 96 sample inlets. IFC controller (Fluidigm) were used to prime 96.96 dynamic arrays IFC Chip (Fluidigm) with control line fluid. 5 µL of each assay and sample mix was then transferred into the appropriate inlets of the primed chip and loaded with the IFC controller. After loading, the chip was placed in the Biomark instrument for Fluidigm screening at 95 °C for 10 minutes followed by 40 cycles at 95 °C for 15 seconds and 60 °C for 1 minute. The data were analyzed with Real-Time PCR Analysis Software in the BIOMARK instrument (Fluidigm Corporation, CA, USA) using manually defined thresholds. Negative samples were defined as those with Cq values ≥ 35 for each bacterial species. A schematic diagram illustrating the workflow of the Fluidigm assay is shown in Supplementary Fig. 3.

All respective reactions included in the Fluidigm assay were optimized according to the MIQE guidelines. Briefly, Standard concentration curves, lower limits of detection (LLDs), correlation coefficient (r^2^), amplification efficiency, analytical specificity, intra-assay variation, accuracy and inter-assay variation for each reaction were calculated as described previously^10^.

### Statistical analysis

Statistical analysis was performed with STATA Version 11.0 (Statacorp, Texas, USA). McNemar’s test was used to determine the sensitivities of culture/qPCR and Fluidigm. When analyzed for concordance, using kappa statistics, only common serogroups/types tested by both methods were included. Findings for non-typable (NT) pneumococcus were not compared between culture and qPCR/Fluidigm, as the qPCR and Fluidigm methods were not optimized to detect all pneumococcal serotypes and thus some untested serotypes might be misidentified as NT pneumococcus. Paired student t-tests were used to compare the mean bacterial concentrations detected by qPCR and Fluidigm, following log_10_ transformation of data. Results were considered significant when the p-value was ≤0.05.

### Ethics approval

Ethical consent was obtained from the Medical Human Research Ethics Committee (HREC) of the University of Witwatersrand for the initial enrolment of the cohort [Vaccinated cohort: HREC: 040704, also registered under Clinical trials number NCT00099658] and all methods were performed in accordance with the relevant guidelines and regulations. The HREC also approved further testing of samples (M140907) in this study. At the time of the initial enrolment, written, informed consent had been obtained from the parents/guardians of all the study participants.

## Supplementary information

Supplementary data

## Data Availability

The data that support the findings of this study are available from the corresponding author upon request.
