# Supplementary material for: Performance of the Biomark HD real-time qPCR System (Fluidigm) for the detection of nasopharyngeal bacterial pathogens and Streptococcus pneumoniae typing
Source: Sci Rep. 2019 Apr 24;9:6494. doi: 10.1038/s41598-019-42846-y (PMC6482308; doi:10.1038/s41598-019-42846-y)
Supplement: Supplementary file 1 — Supplementary data [file 41598_2019_42846_MOESM1_ESM.pdf]

Performance of the Biomark HD real-time qPCR System (Fluidigm) for the detection of nasopharyngeal bacterial pathogens and *Streptococcus pneumoniae* typing.

Courtney P. Olwagen[1, 2, *], Peter V. Adrian[1, 2], and Shabir A. Madhi[1, 2#]

[1] Department of Science and Technology/National Research Foundation: Vaccine Preventable Diseases, Faculty Health Sciences, University of the Witwatersrand, Johannesburg, South Africa.

[2] Medical Research Council: Respiratory and Meningeal Pathogens Research Unit, Faculty Health Sciences, University of the Witwatersrand, Johannesburg, South Africa.

***Corresponding author**

Courtney Paige Olwagen

Respiratory and Meningeal Pathogens Research Unit

Chris Hani-Baragwanath Hospital

Phone: +27 11 983 4262

E-mail: olwagenc@rmpru.co.za

# Alternative corresponding author

Shabir A. Madhi

Professor of Vaccinology, University of Witwatersrand

Medical Research Council: Respiratory and Meningeal Pathogens Research Unit

DST/NRF Research Chair: Vaccine Preventable Diseases

Tel no: +27-11-938-2515

Fax no: 086-582-7830

Email: madhis@rmpru.co.za

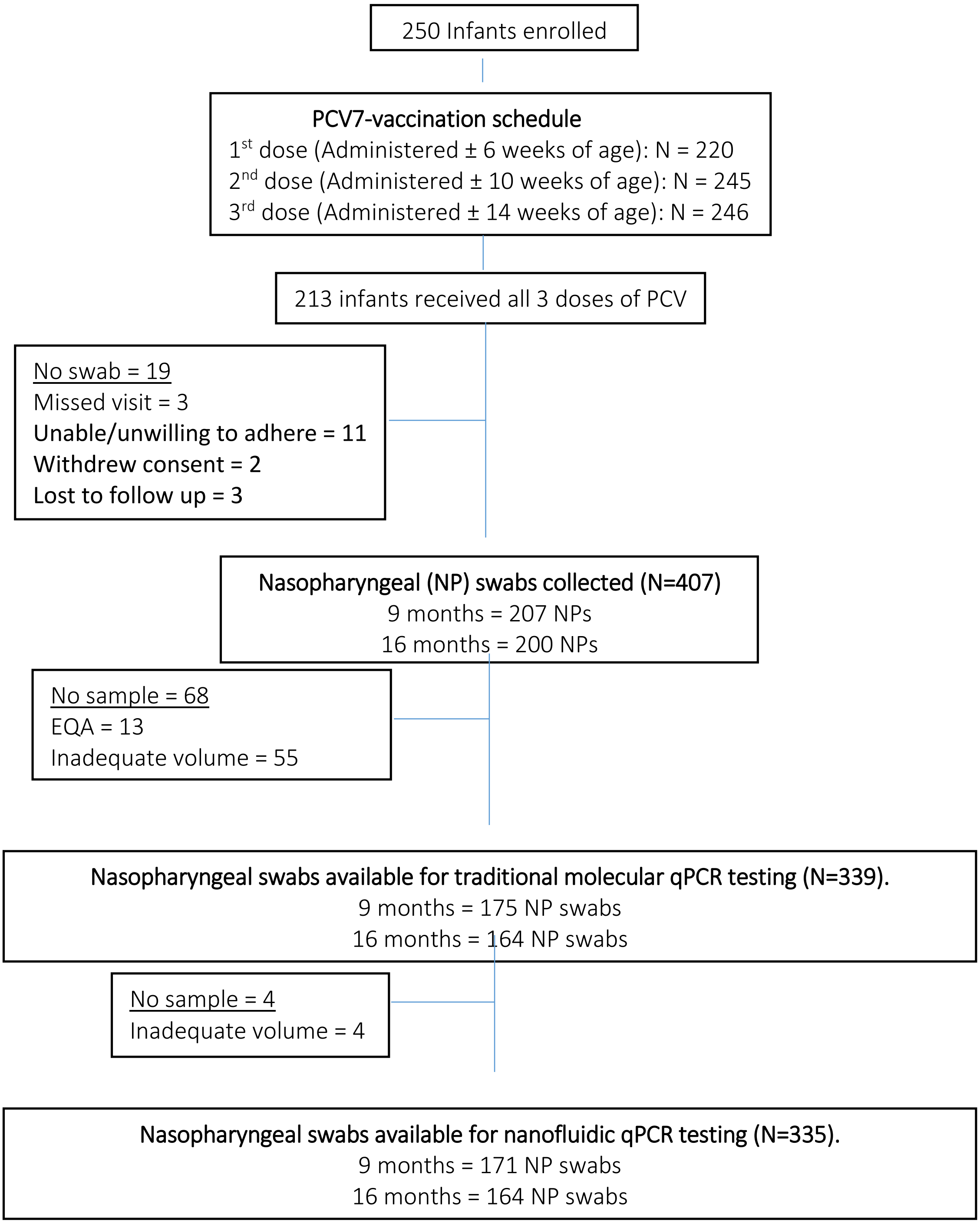

Supplementary Figure 1: Flow diagram of the study population.

Diagram indicating the number of children initially enrolled in the PCV-vaccinated cohort of HIV-uninfected children, as well as the number of nasopharyngeal (NP) swabs available for subsequent traditional molecular qPCR analysis and Biomark HD system (Fluidigm). PCV-vaccinated participants were excluded from analysis if they did not receive all three doses of the pneumococcal conjugate vaccine (PCV) within protocol-defined window periods. The number of NP swabs available for molecular testing was defined by whether there was an adequate volume of sample remaining. EQA, samples were used for external quality assessment.

Supplementary Table 1: Performance of Fluidigm.

Bacterial target	Efficiency (%)	Coefficient correlation (r2)	Limit of detection (copies/PCR)	Intra-assay variation	Accuracy	Inter-assay variation
Streptococcus pneumoniae	104	0.99	10	0.011	0.028	0.045
Haemophilus influenzae	96	0.99	10	0.05	-0.062	0.09
Haemophilus influenzae type A	97	0.99	10	0.031	-0.025	0.038
Haemophilus influenzae type B	91	0.99	10	0.008	-0.078	0.041
Haemophilus influenzae type C	104	0.99	10	0.013	0.065	0.032
Haemophilus influenzae type D	101	0.99	10	0.002	0.083	0.03
Haemophilus influenzae type E	105	0.98	100	0.014	0.039	0.009
Haemophilus influenzae type F	93	0.99	10	0.03	0.05	0.068
Moraxella catarrhalis	97	0.99	10	0.02	-0.057	0.015
Staphylococcus aureus	96	0.99	10	0.007	0.004	0.049
Neisseria lactamica	100	0.99	10	0.063	0.023	0.026
Neisseria meningitidis	91	0.99	10	0.029	-0.049	0.13
ptxs1	99	0.99	10	0.025	-0.081	0.018
HIS1001	104	0.99	10	0.045	-0.071	0.131
PIS1001	91	0.99	10	0.009	0.068	0.021
IS481	99	0.98	100	0.042	0.013	0.12
Streptococcus pyogenes	105	0.99	10	0.018	-0.069	0.09
Streptococcus pneumoniae serotype 1	93	0.99	10	0.011	0.034	0.013
Streptococcus pneumoniae serotype 3	93	0.99	10	0.002	-0.049	0.05
Streptococcus pneumoniae serotype 4	96	0.99	10	0.002	0.06	0.023
Streptococcus pneumoniae serotype 5	105	0.99	100	0.012	0.055	0.041
Streptococcus pneumoniae serogroup 6A/B/C/D	94	0.99	100	0.011	-0.059	0.029
Streptococcus pneumoniae serogroup 6C/D	91	0.99	100	0.013	-0.026	0.047
Streptococcus pneumoniae serogroup 7A/F	98	0.99	10	0.008	-0.054	0.034
Streptococcus pneumoniae serotype 7C	90	0.99	10	0.027	-0.068	0.015
Streptococcus pneumoniae serotype 8	101	0.99	10	0.002	0.104	0.019
Streptococcus pneumoniae serogroup 9A/L/N/V	96	0.99	10	0.006	-0.059	0.016
Streptococcus pneumoniae serotype 10A	90	0.99	10	0.083	-0.001	0.005
Streptococcus pneumoniae serogroup 11A/B/C/D/F	93	0.99	10	0.027	0.011	0.021
Streptococcus pneumoniae serogroup 12ABF/44/46	89	0.99	10	0.003	0.045	0.029
Streptococcus pneumoniae serotype 13	96	0.99	100	0.011	-0.067	0.039
Streptococcus pneumoniae serotype 14	91	0.99	10	0.009	-0.027	0.027
Streptococcus pneumoniae serogroup 15A/B/C/F	102	0.99	10	0.078	-0.052	0.083
Streptococcus pneumoniae serogroup 16F	105	0.99	10	0.011	-0.033	0.025
Streptococcus pneumoniae serotype 17F	92	0.99	10	0.01	-0.041	0.01
Streptococcus pneumoniae serogroup 18A/B/C	99	0.99	10	0.014	-0.016	0.09
Streptococcus pneumoniae serotype 19A	90	0.98	10	0.018	0.077	0.025
Streptococcus pneumoniae serogroup 19B/F	98	0.99	10	0.004	0.009	0.021
Streptococcus pneumoniae serotype 20	103	0.99	10	0.014	0.069	0.003
Streptococcus pneumoniae serotype 21	96	0.99	100	0.111	0.083	0.122
Streptococcus pneumoniae serotype 22A/F	99	0.99	10	0.019	-0.067	0.046
Streptococcus pneumoniae serogroup 23A/B/F	103	0.99	10	0.074	-0.081	0.022
Streptococcus pneumoniae serotype 23F	97	0.99	10	0.006	0.02	0.038
Streptococcus pneumoniae serogroup 25A/25F/38	101	0.99	10	0.015	0.012	0.087
Streptococcus pneumoniae serogroup 34/37/17A	101	0.99	10	0.001	-0.079	0.008
Streptococcus pneumoniae serotype 35B	100	0.99	10	0.013	0.005	0.029

Supplementary Table 2: Concordance between culture and Fluidigm for the detection of common of nasopharyngeal pathogens in PCV-vaccinated, HIV-uninfected children.

**Bacteria**	**Culture**	**Fluidigm**	**Kappa**	**Sensitivity**	**Specificity**
S. pneumoniae	201 (60%)	246 (73.4%)	0.59	96%	60%
H. influenzae	183 (54.6%)	232 (69.3%)	0.63	97%	64%
S. aureus	56 (16.7%)	55 (16.4%)	0.86	96%	97%

Supplementary Table 3: Concordance between culture and Fluidigm for the detection of pneumococcal serotypes in PCV-vaccinated, HIV-uninfected children Serotype/serogroup

	Culture	Fluidigm	Kappa	Sensitivity	Specificity
1	0	5(1.5%)	0	ND	98%
3	1(0.3%)	1(0.3%)	1	100%	100%
4	1(0.3%)	4(1.2%)	0.4	ND	100%
5	0	13(3.9%)	0	ND	98%
6A/B	31(8.2%)	31(8.2%)	0.93	94%	99%
6C/D	0	2(0.6%)	0	0%	99%
7A/F	0	1(0.3%)	0	0%	99%
7C	4(1.2%)	6(1.8%)	0.8	100%	99%
8	2(0.6%)	4(1.2%)	0.66	100%	99%
9A/L/N/V	8(2.4%)	8(2.4%)	1	100%	100%
10A	5(1.5%)	6(1.8%)	0.54	60%	99%
11A/B/C/D/F	5(1.5%)	9(2.7%)	0.56	80%	98%
12AB/44/46	2(0.6%)	5(1.5%)	0.57	100%	99%
13	6(1.8%)	6(1.8%)	1	100%	100%
14	4(1.2%)	7(2.1%)	0.72	100%	99%
15	13(3.9%)	14(4.2%)	0.96	100%	99%
16	5(1.5%)	9(2.7%)	0.71	100%	98%
17F	3(0.9%)	6(1.8%)	0.66	100%	99%
18A/B/C	3(0.9%)	7(2.1%)	0.59	100%	99%
18F	1(0.3%)	0	0	0%	100%
19A	29(8.7%)	36(10.8%)	0.78	90%	97%
19B/F	37(11%)	50(14.9%)	0.77	95%	95%
20	1(0.3%)	1(0.3%)	-	0%	99%
21	3(0.9%)	5(1.5%)	0.75	100%	99%
22A/F	1(0.3%)	2(0.6%)	0.67	100%	99%
23A/B	7(2.1%)	8(2.4%)	0.66	71%	99%
23F	9(2.7%)	15(4.5%)	0.66	89%	98%
25AF/38	0	12(3.6%)	0	ND	96%
34/37/17A	11(3.3%)	13(3.9%)	0.83	91%	99%
33B	1(0.3%)	0	0	0%	100%
35F	1(0.3%)	0	0	0%	100%
35B	0	1(0.3%)	-	ND	99%

Supplementary Table 4: Yield of additional serotypes detected by Fluidigm in relation to being co-carriage with other pneumococcal serotypes

serotype	Number of serotypes co-carried/additional serotypes detected by Fluidigm (%)	Number of dominating colonizing serotype/number of serotypes co-carried (%)
1	4/5(80)	1/4(25)
3	0	0
4	1/3(33)	0/1
5	12/13(92)	0/12
6A/B	1/2(50)	0/1
6C/D	3/4(75)	0/3
7A/F	1/1(100)	0/1
7C	2/2(100)	0/2
8	2/2(100)	0/2
9A/L/N/V	0	0
10A	3/3(100)	1/3(33)
11A/B/C/F	5/5(100)	0/5
12A/12B/12F/44/46	2/3(67)	2/2(100)
13	0	0
14	2/3(67)	0/1
15A/B/C/F	1/1(100)	0/1
16F	3/4(75)	0/3
17F	3/3(100)	0/3
18A/B/C	4/4(100)	1/4(25)
19A	7/10(70)	0/7
19B/F	11/15(73)	1/11(9)
20	1/1(100)	0/1
21	2/2(100)	1/20(50)
22A/F	1/1(100)	0/1
23A/B	2/2(100)	1/2(50)
23F	6/7(86)	1/6(16.7)
25A/25F/38	8/12(67)	1/8(12.5)
34/37/17A	2/3(67)	0/2
35B	1/1(100)	0/1
Total	92/113(81)	10/92(11)

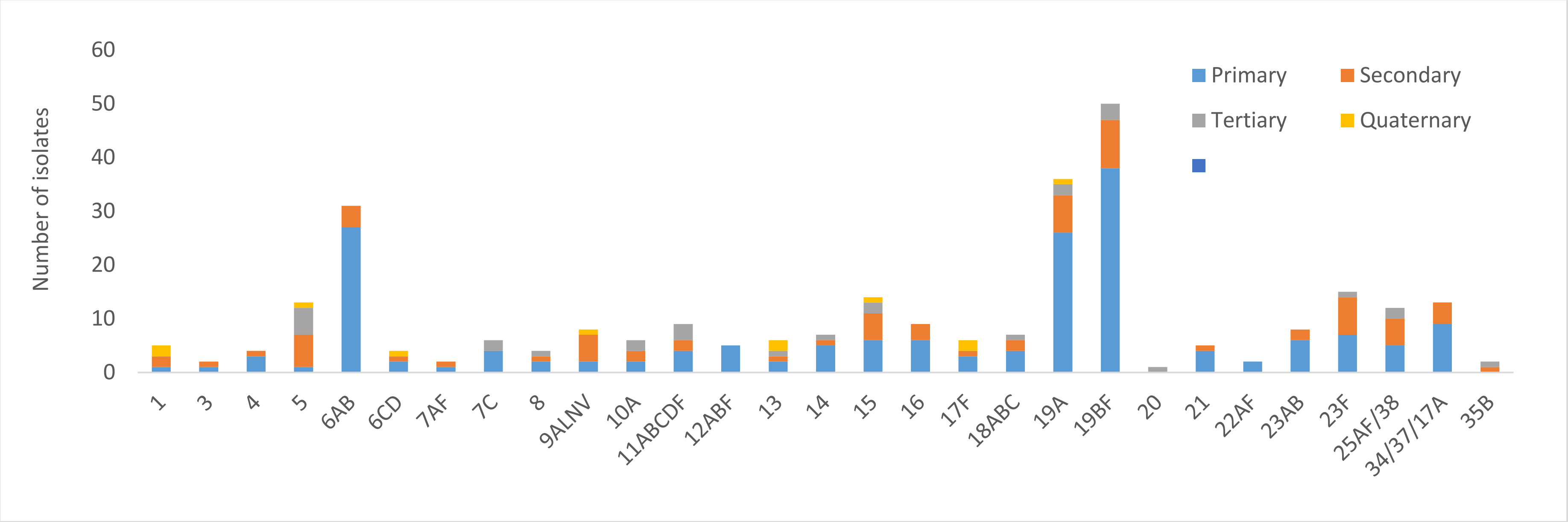

Supplementary Figure 2: Serotype/group specific ranking of multiple pneumococcal carriage

Each isolate of S. pneumoniae was ranked according to its carriage density as determined by Fluidigm to other isolates present in the same sample. Single colonizers were included in the analysis as primary colonizing serotypes.

Supplementary Table 5: Primer pools for Specific Target Amplification (STA) to be used in an initial step (pre-amplification of DNA) for the Fluidigm

**Pool A**	**Pool B**
Streptococcus pneumoniae (*lytA*)	GAPDH
Haemophilus influenzae	*Haemophilus influenzae* type D
Haemophilus influenzae type A	*Haemophilus influenzae* type F
Haemophilus influenzae type B	BexA
Haemophilus influenzae type C	*Staphylococcus aureus*
Haemophilus influenzae type E	*Moraxella catarrhalis*
Neisseria meningitidis	*Streptococcus pyogenes*
Bordetella holmesii	*Neisseria lactamica*
Streptococcus pneumoniae serotype 1	*Bordetella pertussis* and *Bordetella holmesii*
Streptococcus pneumoniae serotype 4	*Bordetella parapertusis*
Streptococcus pneumoniae serotype 5	*Bordetella pertussis*, *Bordetella parapertusis,sp* and *Bordetella bronchiseptica*
Streptococcus pneumoniae serogroup 6A/B/C/D	*Streptococcus pneumoniae* serotype 3
Streptococcus pneumoniae serotype 7C	*Streptococcus pneumoniae* serogroup 6C/D
Streptococcus pneumoniae serotype 8	*Streptococcus pneumoniae* serogroup 7A/F
Streptococcus pneumoniae serogroup 9A/L/N/V	*Streptococcus pneumoniae* serogroup 11A/B/C/D/F
Streptococcus pneumoniae serotype 10A	*Streptococcus pneumoniae* serogroup 12A/12B/12F/44/46
Streptococcus pneumoniae serotype 14	*Streptococcus pneumoniae* serotype 13
Streptococcus pneumoniae serotype 19A	*Streptococcus pneumoniae* serogroup 15A/B/C/F
Streptococcus pneumoniae serogroup 19B/F	*Streptococcus pneumoniae* serogroup 16F
Streptococcus pneumoniae serotype 21	*Streptococcus pneumoniae* serotype 17F
Streptococcus pneumoniae serogroup 23A/B/F	*Streptococcus pneumoniae* serogroup 18A/B/C
Streptococcus pneumoniae serotype 23F	*Streptococcus pneumoniae* serotype 20
Streptococcus pneumoniae serogroup 25A/25F/38	*Streptococcus pneumoniae* serotype 22A/F
Streptococcus pneumoniae serogroup 34/37/17A	*Streptococcus pneumoniae* serotype 35B

Make up 20X TaqMan assays for 1) Specific target amplification and 2) Real-time qPCR using the Biomark HD System (Fluidigm), for each of the 48 reactions listed in Table 4.

1: 18µL Forward primer (100µM stock) + 18µL reverse primer (100µM stock) + 64µL dilution reagent*.

2: 18µL Forward primer (100µM stock) + 18µL reverse primer (100µM stock) + 5µL probe (100µM stock) + 59µL dilution reagent*.

*Dilution Reagent (10 mM Tris-HCl, pH 8.0, 0.1 mM EDTA)

1. SPECIFIC TARGET AMPLIFICATION (STA)

Pool TaqMan Gene Expression Assays

Combine equal volumes of each 20X TaqMan Gene expression Assays into two separate pools, of 24 assays each, as described in Supplementary table 5. Each assay is at a final concentration of 0.2X (180nM).

Pool A
2 µL X 24 TaqMan assays (48ul total)
62 µL dilution reagent *
Total volume = 200ul pool A

Pool B
2 µL X 24 TaqMan assays (48ul total)
62 µL dilution reagent *
Total volume = 200ul pool B

Prepare Sample Pre-Mix

In a DNA-free hood, prepare the sample pre-mix for the reactions for each respective primer pool.

Pool A
1µL Preamp Master Mix (Fluidigm PN 100-5744)
1.25µL Pooled A TaqMan assay mix (0.2X)
1.5µL Water
cDNA 1.25µL
Total volume 5µL

Pool B
1µL Preamp Master Mix (Fluidigm PN 100-5744)
1.25µL Pooled B TaqMan assay mix (0.2X)
1.5µL Water
cDNA 1.25µL
Total volume 5µL

Preform specific target amplification reactions for each sample, for both pools A and B, in a standard thermo-cycler

Cycling conditions include an initial activation at 95ºC for 10 minutes followed by 14 two-step cycles (denaturation at 95ºC for 15 seconds and annealing/extension at 60ºC for 4 minutes).

Combine STA products from pool A and pool B and dilute 1:5 with dilution reagent*

After cycling, dilute the reaction 1:5 by adding 15µL Dilution Reagent to the final 10µL (5µL pool A product + 5µL pool B product) reaction volume for a total volume of 25µL. Assay immediately or store at -20 degrees.

2. REAL-TIME QPCR USING THE BIOMARK HD SYSTEM (FLUIDIGM)

Prime the 96.96 IFC

Inject control line fluid into each accumulator on the IFC and then place the IFC into the Juno. Run script: Prime 96.96 GE.

Prepare 10X Assays

Prepare aliquots of 10X assays (for each of the 48 assays listed in table 4) using volumes described below. Prepare each assay in duplicate to make a total of 96 assays.

Component	Vol. per inlet (µL)	Vol. per inlet with overage (µL)	Vol. for 50 µL stock
20X TaqMan Gene Expression assays	2.5	3	25
2X Assay Loading Reagent (Fluidigm PN 100-7611)	2.5	3	25
Total	5	6	50

Final concentration (at 10X): primers, 9 µM; probe, 2 µM

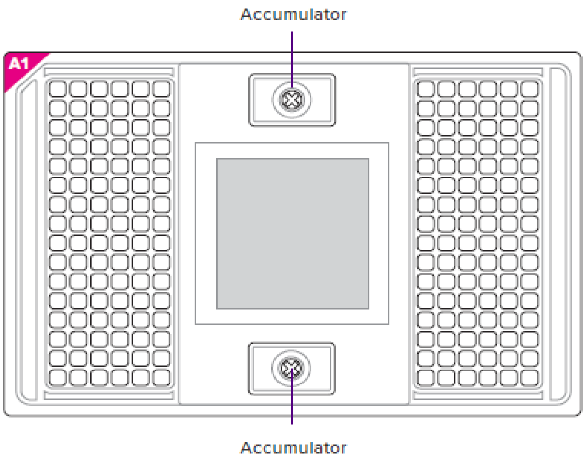

Prepare Sample Pre-Mix and Samples

Component	Vol. per inlet (µL)	Vol. per inlet with overage (µL)	Sample pre-mix for 96.96 (µL)
TaqMan Universal PCR Master Mix (2X) (Life Technologies PN 4304437)	2.5	3.0	360
20X GE Sample Loading Reagent(Fluidigm PN 100-7610)	0.25	0.3	36.0
Pre-amplified cDNA (diluted product from step 1)	2.25	2.7	
Total	5.0	6.0	

In a DNA-free hood, combine the master mix with the 20X GE Sample Loading Reagent in a 1.5 mL sterile tube - enough volume to fill an entire IFC. Vortex to mix and centrifuge briefly. Aliquot 3.3µL of this sample pre-mix for each sample. Remove the aliquots of sample pre-mix from the DNA-free hood and in a DNA sample hood add 2.7µL of sample to each, making a total volume of 6µL in each aliquot. Vortex to mix and centrifuge.

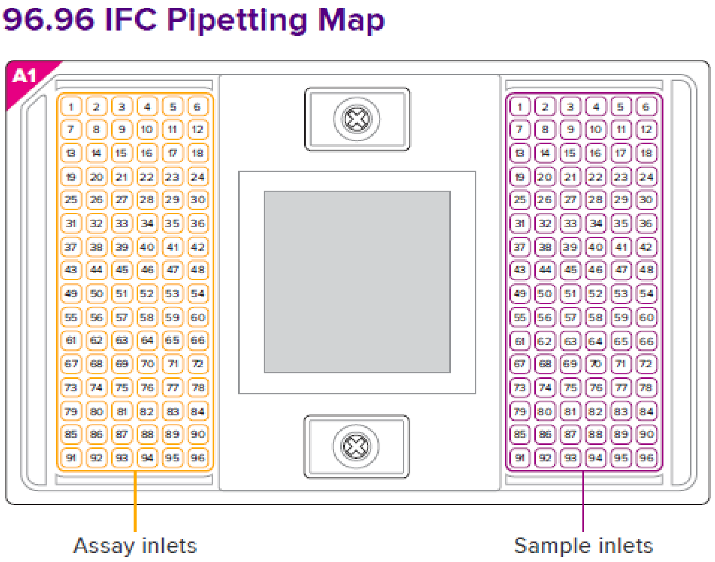

Load the IFC

Vortex thoroughly and centrifuge all assay and sample solutions before pipetting into the IFC inlets. When the prime script has finished, remove the primed IFC from the instrument and pipet 5µL of each assay and each sample into their respective inlets on the IFC as shown in IFC pipetting map. Return the IFC to the instrument and run the load script: Load Mix 96.96 GE.

NOTE: Start IFC run within 1 hour of loading samples.

Collect Real-Time PCR Data

Once load script is finished, remove IFC from the Juno and place in the Biomark HD system for Real-time qPCR.

Supplementary Figure 3: flow diagram illustrating the workflow of the Fluidigm assay
